# Eating Patterns during Pregnancy and Postpartum and Their Association with Diet Quality and Energy Intake

**DOI:** 10.3390/nu14061167

**Published:** 2022-03-10

**Authors:** Carolina Schwedhelm, Leah M. Lipsky, Chelsie D. Temmen, Tonja R. Nansel

**Affiliations:** 1 Social and Behavioral Sciences Branch, Division of Intramural Population Health Research, Eunice Kennedy Shriver National Institute of Child Health and Human Development, National Institutes of Health, Bethesda, MD 20817, USA; carolina.schwedhelm@mdc-berlin.de (C.S.); leah.lipsky@nih.gov (L.M.L.); chelsie.temmen@louisville.edu (C.D.T.); 2 Molecular Epidemiology Research Group, Max-Delbrueck-Center for Molecular Medicine in the Helmholtz Association, 13125 Berlin, Germany; 3 Department of Counseling and Human Development, The University of Louisville, Louisville, KY 40292, USA

**Keywords:** eating frequency, eating regularity, intake timing, pregnancy, postpartum, energy intake, diet quality

## Abstract

This study investigates the relationship between meal-specific eating patterns during pregnancy and postpartum with maternal diet quality and energy intake. Participants in a prospective cohort study completed 24-h dietary recalls three times throughout both pregnancy and 1 year postpartum (*n* = 420). Linear regressions estimated the associations of eating frequency (number of daily main meals and eating occasions), meal and energy regularity (meal skipping and variation of daily energy intake), and intake timing patterns (distribution of energy intake throughout the day, derived using principal component analysis) with daily energy intake and diet quality (Healthy Eating Index-2015, calculated daily and overall, across both pregnancy and postpartum). Eating frequency was positively associated with energy intake and daily diet quality. Irregular meals were associated with lower energy intake in pregnancy but not postpartum and with lower pregnancy and postpartum diet quality. Energy irregularity was not associated with energy intake or diet quality. Higher postpartum diet quality was associated with a morning energy intake pattern (versus late morning/early afternoon or evening). Differences in these associations between pregnancy and postpartum suggest that efforts to support optimal energy intake and diet quality by modifying eating patterns may require specific strategies for pregnancy and postpartum.

## 1. Introduction

Maternal dietary intake among U.S. women during pregnancy and postpartum does not meet minimum recommendations for whole grains/fiber [1,2,3,4,5], vegetables [1,2,3,4], fruits [1,3,4], and dairy [1,3] and exceeds recommendations for total fat [2], refined grains [1,3], sodium [1,3], and empty calories [3]. Furthermore, approximately half of U.S. pregnant women exceed recommended gestational weight gain [6] and only about half of women return to pre-pregnancy weight within 1–3 years [7]. Poor diet quality during pregnancy [8,9] and postpartum [10], excessive gestational weight gain [11,12], and weight retention [13] are risk factors for numerous adverse maternal and child health outcomes. Identifying modifiable influences on diet quality and energy intake during pregnancy and postpartum could strengthen efforts to decrease morbidity and mortality in this population.

The temporal organization of eating occasions within and across days (i.e., eating patterns) may influence diet quality and energy intake [14] via several behavioral and appetitive pathways [14,15,16,17,18,19,20]. Key aspects of eating patterns that have been investigated include frequency (i.e., the number of meals or eating occasions throughout the day), regularity (i.e., day-to-day consistency of the number and spacing of meals, or of total energy intake), and food intake timing (i.e., the time of eating occasions and proportion of daily intake consumed at that time). Greater eating frequency has been positively related to diet quality [21,22] and both positively and inversely related to energy intake [15,22,23] in non-pregnant samples. Regularity of meals and total daily energy intake across days is positively associated with diet quality [24,25,26,27,28], but findings have been mixed for the association of meal regularity with energy intake [16,25,29]. Studies using data-driven methods to derive patterns of food intake timing indicate that both late eating [30,31,32] and grazing [23] (i.e., several small eating occasions throughout the day) are associated with lower diet quality and higher energy intake.

Although evidence suggests that food intake patterns may be influenced by the physiological and behavioral demands of pregnancy and postpartum [33,34,35,36,37], few studies have examined the relationship of food intake patterns with diet quality or energy intake during this developmental period. Among the few studies of food intake patterns in pregnant and postpartum women, relations of meal frequency with total energy intake have been inconsistent [38,39,40], and findings of an inverse association of late-night eating with energy intake [41] and a positive association of meal skipping with diet quality [42] are contrary to findings in the general population. The aim of this study is to examine associations between eating frequency, regularity, and timing and diet quality and energy intake during pregnancy and postpartum.

## 2. Materials and Methods

### 2.1. Design and Participants

The Pregnancy Eating Attributes Study (PEAS) was a prospective study of women aged 18–44 years enrolled at ≤12 weeks gestation and followed up one-year postpartum (clinicalTrials.gov identifier: NCT02217462). Participants were recruited from two obstetrics clinics at the University of North Carolina (UNC) at Chapel Hill, North Carolina, United States from 2014 to 2016. The primary study aim was to investigate the relationship of reward-related eating with maternal diet and weight change during pregnancy and postpartum. Women were assessed each pregnancy trimester (at ≤12, 16–22, and 28–32 weeks gestation) and postpartum at 4–6 weeks, 6 months, and 12 months. The data collection was completed in June 2018 [43]. Additional inclusion criteria were anticipating uncomplicated singleton pregnancy, willing to undergo study procedures and provide informed consent for her participation and assent for the baby’s participation, BMI ≥ 18.5 kg/m^2^, ability to complete self-report assessments in English, access to the internet with email, planning to deliver at UNC Hospital, and planning to remain in the geographical vicinity for 1 year following delivery. Exclusion criteria were multiple pregnancy, pre-existing diabetes, participant-reported eating disorder, and any medical or psychosocial conditions contraindicating participation in the study. Additional study details are available elsewhere [43]. The study was approved by the UNC Institutional Review Board. All participants provided signed informed consent.

### 2.2. Measures

#### 2.2.1. Dietary Intake

Participants were prompted to complete one (but were allowed to provide more) 24-h dietary recall per study visit, which they accessed through a study website. Dietary recalls were collected using the Automated Self-Administered 24-Hour Recall (ASA24), a web-based tool developed by the National Cancer Institute [44], which prompts participants to report all foods consumed structured by eating occasion, which may include “breakfast”, “brunch”, “lunch”, “dinner”, “supper”, “snack”, “just a drink”, and “just a supplement” (i.e., vitamins, minerals, herbals and other dietary supplements, including prescription supplements), and time of day. Data entered into the ASA24 were coded using the USDA’s Food and Nutrient Database for Dietary Studies (FNDDS) [45], from which nutrients and USDA Food Patterns Equivalents Database food groups were computed. Dietary recalls indicating <600 kcal/day were considered implausible and were excluded from analysis. Recalls indicating >4500 kcal/day were reviewed individually and were determined to reflect a plausible intake and were retained for analysis. Eating occasions reported as supper were recoded as dinner, and where brunch but no breakfast was reported, brunch was recoded as breakfast. Eating occasions of >0 kcal reported as “just a drink” and “just a supplement” were recoded as “snack”. The Healthy Eating Index-2015 (HEI), calculated from the USDA Food Patterns Equivalents Database food groups computed from the ASA24, is an indicator of diet quality and reflects adherence to the 2015–2020 Dietary Guidelines for Americans (DGA) [46]. The HEI ranges from 0 to 100, with higher scores indicating a healthier diet, and consists of the sum of 9 adequacy components (total fruit, whole fruit, total vegetables, greens and beans, whole grains, dairy, total protein foods, seafood and plant proteins, and fatty acids) and 4 moderation components (refined grains, sodium, added sugars, and saturated fats), scored based on energy-adjusted intakes (per 1000 kcal) [47]. Little change in diet quality across pregnancy trimesters has been reported in previous studies [48,49]; therefore, in addition to HEI scores calculated by dietary recall day, overall pregnancy and postpartum HEI were calculated by pooling all dietary recalls from pregnancy and postpartum, respectively, using the simple HEI scoring algorithm per person [50].

#### 2.2.2. Demographic, Medical, and Lifestyle Data

Participants reported their demographic information at baseline, including marital status, household composition, income, education, race/ethnicity, and employment status. Income-to-poverty ratio was calculated from the participant-reported household size and household income [51]. Age and parity were obtained from medical records. Early pregnancy BMI (kg/m^2^) was calculated from the height and weight values measured at the baseline visit. Lifestyle data including breastfeeding, physical activity, and eating less due to nausea were obtained by self-report through online questionnaires completed within each study visit window. Breastfeeding duration was calculated from participant-reported current and previous breastfeeding at each visit and includes any breastfeeding from the breast and from the bottle. Participants reported frequency and type of physical activity as times per week. Activities were categorized according to intensity, based on previously published activity-specific metabolic equivalents of task (MET) intensities [52,53].

### 2.3. Statistical Analysis

#### 2.3.1. Eating Frequency

Main meal frequency was examined as the number/day of main meals (i.e., breakfast, lunch, dinner). If a participant reported more than one of a given meal type within a single day, the additional meal contributed toward the total main meal frequency. Eating occasion frequency was examined as the number of eating occasions ≥50 kcal per day. Mixed linear regression with restricted maximum likelihood (REML) estimation examined the relationship of frequency of all eating occasions and main meals with daily energy intake and daily HEI, in separate models for pregnancy and postpartum. A random intercept for participants was used to account for repeated measures (i.e., recall or visit specific variables).

#### 2.3.2. Eating Regularity

Participants with only one dietary recall per pregnancy or postpartum were excluded from all eating regularity analyses. Meal regularity was examined as a binary variable (regular meal-eating versus meal skipping), whereas regular meal-eating was defined as consuming all main meals (breakfast, lunch, dinner) on all available recalls, and meal skipping as skipping any main meal on any available recall. Energy irregularity was examined using a previously published energy irregularity score [54], calculated as the absolute difference of the daily energy intake from each dietary recall and the mean daily energy intake from all available recalls, multiplied by 100 and averaged over all available recalls. A higher score indicates higher variation in energy intake across days. Eating occasions < 50 kcal were excluded from meal regularity but not from energy regularity analyses. Linear regression with full information maximum likelihood (FIML) estimation examined the relationship of meal and energy regularity with mean daily energy intake and overall HEI in separate models for pregnancy and postpartum.

#### 2.3.3. Intake Timing

**Figure 1:**
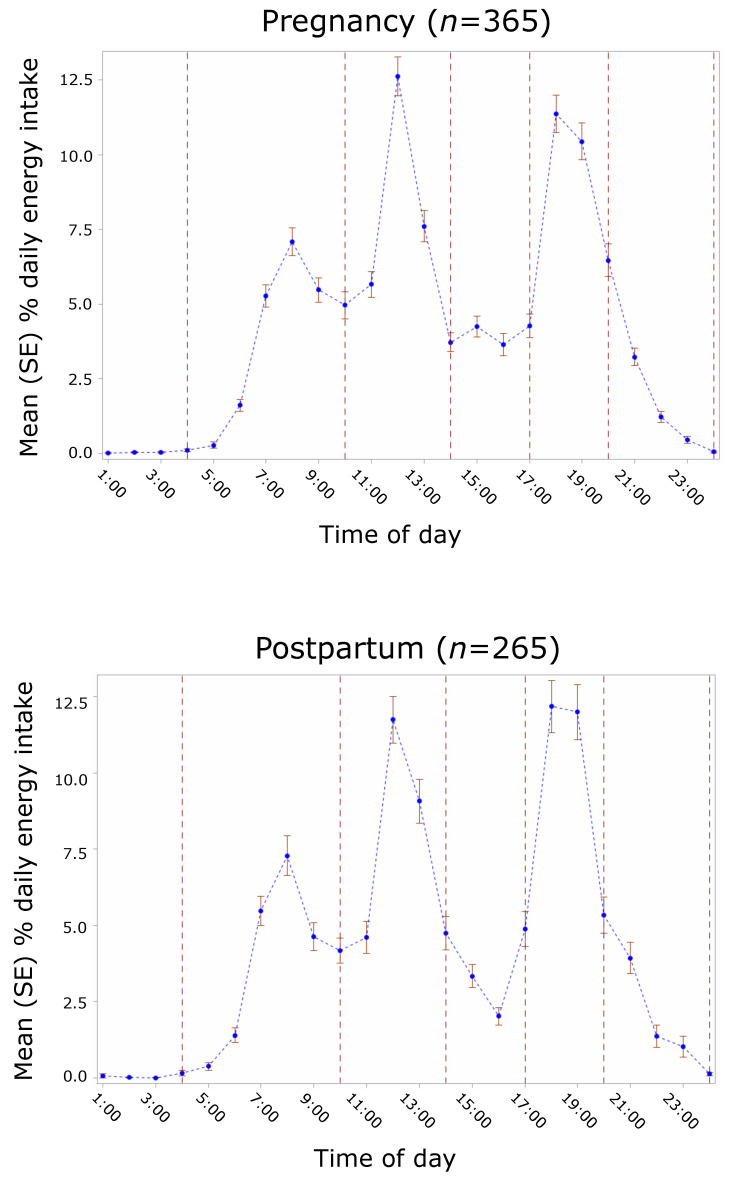
Intake timing in the Pregnancy Eating Attributes Study (PEAS) and time windows for PCA pattern identification. Blue dots with error bars show the mean percent daily energy intake at each hour with corresponding standard errors. Vertical dashed lines indicate the time windows used for PCA pattern identification based on the observed intake pattern by time of day.

The distribution patterns of mean energy intake (temporal eating patterns) throughout the day were identified by factor analysis using principal component analysis (PCA) with varimax rotation and using the covariance matrix. PCA is a data reduction technique that produces linear combinations (i.e., factor loadings) of the input variables (i.e., components or patterns) in a way that explains the highest possible variance [55]. The criteria used for the number of retained patterns were an eigenvalue ≥1 and inspection of the scree plot. Patterns were derived separately for pregnancy and for postpartum data, and variables used for pattern identification were percent of mean daily energy intake within five time windows, based on the identified peaks of consumption in the data: 4:00 to 10:00, 10:01 to 14:00, 14:01 to 17:00, 17:01 to 20:00, and 20:01 to 24:00 h. The time window of 00:00 to 3:59 was excluded from analysis due to almost exclusively null values (Figure 1). All eating occasions > 0 kcal were considered for pattern identification and subsequent analyses. Missing times of eating occasion were imputed using the overall mean time by type of eating occasion (breakfast, lunch, dinner, snack). A score was derived for each participant for each pattern in pregnancy and each pattern in postpartum by summing participants’ mean percent daily energy intake at each time window weighted by the PCA factor loading. Higher scores reflect a higher adherence to the patterns. Input variables with factor loadings of ≥0.3 on a pattern were considered to be important contributors to that pattern. The relationship of intake timing with mean daily energy intake and overall HEI score was examined using linear regression with full information maximum likelihood (FIML) in separate models for pregnancy and postpartum. Pattern score adherence variables for all retained patterns were included simultaneously in the models.

All adjusted models were controlled for income (income-poverty ratio), race/ethnicity (white, non-Hispanic; black, non-Hispanic; Hispanic or Latino; Asian, other or multi-race), education (high school or less, some college, associate’s degree, bachelor’s degree or higher), recall day (week/weekend), and moderate/vigorous physical activity frequency (times/week per study visit window). Additionally, pregnancy models adjusted for eating less due to nausea (never, sometimes, often per study visit window), and postpartum models adjusted for breastfeeding (yes/no per study visit window). Selection of adjustment variables was identified based on the literature [56] and on variables hypothesized to be causally related to both the independent and dependent variables but not along the causal pathway. Recall/visit-specific covariates were collapsed into one observation per participant for inclusion in linear regression models (i.e., eating regularity and intake timing) as follows: proportion of recalls on weekend days, mean moderate/vigorous physical activity frequency (mean times/week), highest reported frequency of eating less due to nausea (1 = never, 2 = sometimes, 3 = often) reported (pregnancy models), and total breastfeeding duration in months (postpartum models). The 95% confidence intervals (95% CI) were reported, and α < 0.05 was used to indicate statistical significance. Data analysis was conducted in SAS (Version 9.4, Enterprise Guide 7.1, SAS Institute Inc., Cary, NC, USA).

## 3. Results

**Figure 2:**
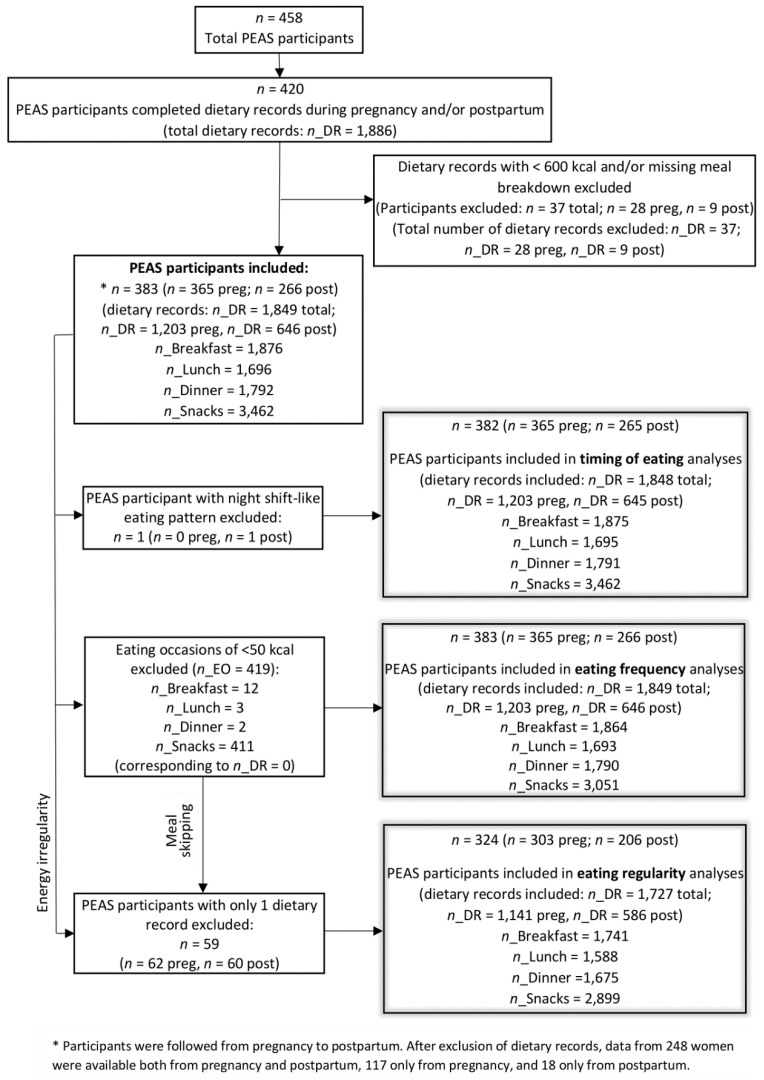
Flow diagram of PEAS participants for analysis in the present study. *n*, number of participants; *n*_DR, number of dietary records; *n*_EO, number of eating occasions; preg, participants in pregnancy; post, participants in postpartum.

Of 458 women enrolled, 420 women completed dietary recalls during pregnancy and/or postpartum. After excluding dietary recalls of <600 kcal and one with missing meal information, dietary data from 365 participants in pregnancy and 266 participants in postpartum (of which 248 have dietary data both in pregnancy and in postpartum) were available for analysis (Figure 2), corresponding to 1203 and 646 dietary records, respectively. Participants were on average 31 years old and about half had a normal BMI. Most women were married and had an advanced degree, and the majority were non-Hispanic white. Participants completed approximately 3 dietary recalls each in pregnancy and postpartum, consumed approximately 2000 calories per day, and had a mean daily HEI score of approximately 55. Participants consumed approximately 3 main meals per day and about a third of the participants skipped a main meal in one or more of the dietary recalls. The time windows with the highest contribution to daily energy intake were 10:00–14:00 h and 17:00–20:00 h, with nearly a third of the daily energy intake at each time window (Table 1).

### 3.1. Eating Frequency

#### 3.1.1. Daily Energy Intake

Main meal frequency was positively associated with daily energy intake in pregnancy and postpartum. In adjusted models, one additional main meal was associated with an additional 300.7 kcal (95% CI: 199.3–402.0) in pregnancy and additional 243.3 kcal (95% CI: 86.1–400.6) in postpartum. The positive association remained, albeit slightly attenuated, when including all eating occasions > 50 kcal; in adjusted models, an additional eating occasion was associated with an additional 161.6 (95% CI: 129.6–193.5) kcal in pregnancy and an additional 146.4 kcal (95% CI: 91.4–201.4) in postpartum (Table 2).

#### 3.1.2. Daily HEI

Similarly, main meal frequency was positively associated with daily HEI in pregnancy and postpartum; however, in postpartum the association was no longer statistically significant after adjusting for covariates. In adjusted models, one additional main meal was associated with a 2.9 (95% CI: 0.8–5.0) higher HEI in pregnancy and a 3.1 (95% CI: −0.1 to 6.2) higher HEI in postpartum. When examining all eating occasions ≥50 kcal (including main meals), positive associations of eating frequency with HEI were significant in both unadjusted and adjusted models. In adjusted models, an additional eating occasion was associated with a 1.9 (95% CI: 1.2–2.5) higher HEI in pregnancy and a 2.5 (95% CI: 1.4–3.6) higher HEI in postpartum (Table 2).

### 3.2. Eating Regularity

After exclusion of participants with only one dietary recall, data from 303 participants in pregnancy and 206 participants in postpartum were used for analysis, corresponding to 1141 and 586 dietary records, respectively (Figure 2).

#### 3.2.1. Mean Energy Intake

In adjusted models, skipping >1 meals in any of the available recalls was associated with consuming 243.4 fewer kcal per day (95% CI: −384.4 to −102.3) compared to skipping 0 meals (i.e., regular meal eating) in pregnancy, but there was no association in postpartum. Conversely, energy irregularity was not associated with mean energy intake in either pregnancy or postpartum (Table 3).

#### 3.2.2. Overall HEI

In adjusted models, skipping >1 meals in any of the available recalls was associated with a 4.2 (95% CI: −6.7 to −1.6) lower HEI in pregnancy and a 6.2 (95% CI: −9.7 to −2.7) lower HEI in postpartum compared to skipping 0 meals (i.e., regular meal eating). Energy irregularity across days was also inversely associated with overall HEI in pregnancy and postpartum; however, these associations were no longer statistically significant after adjusting for covariates (Table 3).

### 3.3. Intake Timing

After exclusion of one participant with a night shift-like eating pattern, data from 365 participants in pregnancy and 265 participants in postpartum were used for analysis, corresponding to 1203 and 645 dietary records, respectively (Figure 2). Percent energy intake was highest at 12:00 (12.62%), followed by 18:00 (11.36%) in pregnancy, while in postpartum energy intake was slightly higher at 18:00–19:00 (12.17% and 11.99%, respectively), followed by 12:00 (11.74%). The third highest peak of daily energy contribution was at 8:00 in both pregnancy (7.09%) and postpartum (7.28%). While there was a small peak in the late afternoon in pregnancy (4.25% at 15:00), there was a small depression in postpartum (2.02% at 16:00). From 21:00–24:00, contribution to daily energy was low and steadily decreased across time in both pregnancy and postpartum. The contribution was negligible from 1:00–4:00 (time window excluded from PCA pattern identification) (Figure 1).

#### 3.3.1. PCA Patterns

**Figure 3:**
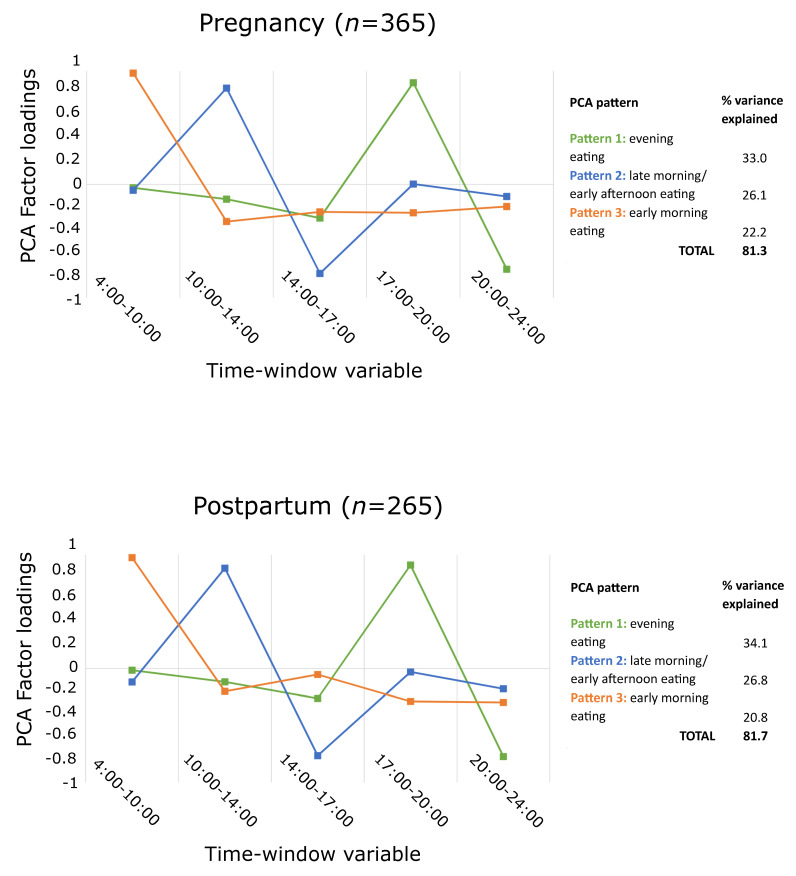
Identified PCA patterns for intake timing.

Three patterns were retained both in pregnancy and in postpartum, explaining 81.3% and 81.7% of the variance, respectively (Figure 3). Intake timing patterns were consistent in pregnancy and in postpartum: the pattern explaining the most variance loaded highly in the time window 17:00–20:00 and was labeled as evening eating, the pattern explaining the second highest variance loaded highly in the time window 10:00–14:00 and was labeled as late morning/early afternoon eating, while the third pattern loaded highly in the time window 4:00–10:00 and was labeled as early morning eating.

#### 3.3.2. Mean Energy Intake

Higher adherence to an early morning eating pattern was associated with a lower mean energy intake in pregnancy, but there was no association in postpartum. Although the association was statistically significant in pregnancy, a 1 SD higher adherence to an early eating pattern was associated with a clinically insignificant effect size of 1.4 kcal/d (95% CI: −2.5 to −0.3) lower energy intake. No other associations were observed for the other intake timing patterns (Table 4).

#### 3.3.3. Overall HEI

Higher adherence to an early morning eating pattern was associated with higher overall HEI in pregnancy and in postpartum. In pregnancy, this association did not reach statistical significance. In postpartum, a 1 SD higher adherence to an early eating pattern was associated with a 1.4 (95% CI: 0.2–2.6) higher HEI. Additionally, an evening eating pattern was positively associated with overall HEI in postpartum in the unadjusted model; there was no association after adjustment for covariates (Table 4).

## 4. Discussion

These findings indicate that associations of eating patterns with energy intake and diet quality were somewhat different in pregnancy versus postpartum in U.S. women. In both periods, greater eating frequency was associated with higher daily energy intake and diet quality and skipping meals were associated with lower diet quality. However, in pregnancy (but not postpartum), skipping meals was associated with a lower energy intake and consuming more calories in the morning (morning eating pattern) was associated with lower energy intake. Furthermore, higher adherence to a morning eating pattern was associated with higher HEI in postpartum only. Energy irregularity was not associated with either daily energy intake or diet quality in either period.

The positive association of eating frequency with energy intake in pregnant and postpartum women is consistent with one study of pregnant women from North Carolina [38] and with one study of postpartum women with overweight and obesity from Sweden [39]; in contrast, our findings were inconsistent with one previous study that reported no association among pregnant women with overweight and obesity from Ireland [40]. Our finding of a positive association of intake frequency with diet quality in pregnancy is also consistent with a study of pregnant women from Brazil [57], although the effect estimate was much smaller than that reported here (0.27 versus 1.9 for eating occasions in pregnancy). Differences in the findings between studies could be attributable to differences in the characteristics of the study populations and dietary intake assessment period (first trimester only), as well as to different meal definitions (>50 kcal, >15 min apart) and diet quality outcomes (BHEI-R, HEI-2005 adapted to the Brazilian population), although mean intake frequency and diet quality during pregnancy were comparable.

Few studies have examined relations of eating regularity with energy intake and diet quality in pregnant and postpartum women. While one study in Japanese pregnant women similarly found that breakfast skipping (>2 times/week) was associated with lower daily energy intake [58], two other studies reported null associations of indicators of meal regularity with energy intake and diet quality [40,42]. Given the limited evidence and large sociodemographic heterogeneity between study samples, more research using more similar methods is needed to understand the impact of eating regularity on overall energy intake and diet quality.

While the lack of an association of early morning eating with diet quality in pregnant women is inconsistent with one study of pregnant women from Brazil [57] reporting a positive association, our finding of a positive association in postpartum could not be compared to other studies given the limited evidence in this developmental period. Surprisingly, our findings of the relationship of early morning eating pattern with energy intake and diet quality differed between pregnancy and postpartum. This may be partly explained by a modestly reduced relevance of an early morning pattern in postpartum versus pregnancy (20.8% versus 22.2% explained variance in pregnancy), which may reflect changes in the wake-sleep cycle in postpartum due to demands of infant care [59]. Other determinants of eating behavior, such as weight control, may also play a role; while weight control is an important determinant of eating behavior both in pregnancy and postpartum [37], it may have a stronger influence on healthy eating behavior in postpartum, when cravings and aversions may have subsided [60] and when motivation for returning to pre-pregnancy weight may have set in [61,62]. Findings regarding the association of nighttime eating and total daily energy intake in pregnancy are inconsistent across studies. While we found no association of an evening eating pattern with energy intake, while inverse associations have been observed in studies in Brazil and Singapore [41,63] and a positive association has been observed in a study in Norway [64]. Possible explanations for these inconsistencies include differences in the exposure definition (i.e., based on meal labels or using different thresholds of daily energy intake within slightly different time windows) and sociodemographic differences between study populations (e.g., inclusion of shift workers), which limit comparability.

Our results suggest potential eating pattern intervention targets to improve diet quality and appropriate energy intake during pregnancy and postpartum, such as examining whether encouraging more frequent meals and snacks and less meal skipping may lead to improvements in diet quality. However, our findings regarding eating frequency and meal regularity need to be interpreted with caution; while regular meals and higher eating frequency were associated with higher diet quality, they were also associated with higher energy intake. Recommendations targeting a lower energy intake could inadvertently lower diet quality; shifting intake toward nutrient-dense foods could ensure that diet quality is not adversely impacted. Another approach to support diet quality and lower total energy intake in pregnancy and postpartum could be recommending consuming a higher proportion of the day’s energy intake in the morning.

Findings from this study should be interpreted in consideration of the strengths and limitations. While there is no standard measure for eating frequency, regularity, and timing, we examined two different measures of eating frequency and regularity and used PCA to identify distribution patterns of energy intake throughout the day in our sample, capturing multiple aspects of intake timing. Most studies on eating patterns in this developmental period focus on pregnancy, with scant research in postpartum. While this study is the first to examine many of these relations in postpartum, and the first to examine the construct of energy irregularity in pregnancy and postpartum, we could not examine differences across pregnancy trimesters or different times in postpartum, as only one dietary recall was available per participant at each visit on average. Our findings may also have limited generalizability, since the sample comes from a single geographic region in the U.S. Finally, due to the observational nature of our study, causality cannot be inferred and experimental studies are needed to ascertain the degree to which eating frequency, regularity, and timing can be manipulated during pregnancy and postpartum, and whether these modifications lead to changes in diet quality and total energy intake.

## 5. Conclusions

In summary, our findings suggest differences in the underlying processes driving these associations between pregnancy and postpartum and highlight a need to better understand pregnancy- and postpartum-specific determinants of eating patterns to more effectively support optimal energy intake and diet quality.

## Figures and Tables

**Table 1:** Sample characteristics of participants with dietary recall data in pregnancy and/or postpartum in PEAS.

Subject Characteristics	Mean ± SD or N (%)		
	Overall *n* = 383 ^1^	Pregnancy *n* = 365	Postpartum *n* = 266
**Sociodemographic**			
Age, y	30.8 ± 4.6	30.8 ± 4.6	30.9 ± 4.3
Baseline BMI group			
Normal weight, 18.5 ≤ BMI < 25	191 (49.9)	186 (51.0)	133 (50)
Overweight, 25 ≤ BMI < 30	99 (25.9)	97 (26.6)	71 (26.7)
Obese, 30 ≤ BMI	93 (24.3)	82 (22.5)	62 (23.3)
Married/living with partner	322 (91.7)	315 (92.1)	237 (94.1)
Income-poverty ratio	3.9 ± 1.9	3.9 ± 1.9	4.1 ± 1.9
Education			
High school or less	29 (8.3)	27 (7.9)	16 (6.4)
Some college	36 (10.3)	35 (10.2)	18 (7.1)
Associate’s degree	29 (8.3)	28 (8.2)	19 (7.5)
Bachelor’s degree or higher	257 (73.2)	252 (73.7)	199 (79.0)
Race/Ethnicity			
White, non-Hispanic	251 (68.8)	247 (71.0)	184 (71.0)
Black, non-Hispanic	53 (14.5)	45 (12.9)	31 (12.0)
Hispanic or Latino	29 (8.0)	26 (7.5)	22 (8.5)
Asian, other or multi-race	32 (8.8)	30 (8.6)	22 (8.5)
**Dietary intake**			
Number of dietary records per participant	-	3.3 (1.7)	2.4 (1.0)
Total daily energy intake, kcal/d	-	2047.2 (657.4)	1995.9 (642.5)
Daily HEI score (0–100) ^2^	-	54.7 (11.2)	55.8 (12.2)
Eating frequency, main meals/d ^3^	-	2.9 (0.3)	2.8 (0.3)
Eating frequency, all eating occasions/d ^4^	-	4.6 (1.2)	4.3 (1.0)
Meal regularity, meal skipping pattern ^5^	-	102 (33.7)	57 (27.7)
Energy irregularity score	-	16.7 (8.9)	16.0 (9.9)
Percent daily energy intake at predefined time windows	-		
04:00–10:00 h	-	23.0 (10.8)	22.0 (10.4)
10:00–14:00 h	-	30.2 (11.6)	30.1 (12.6)
14:00–17:00 h	-	10.8 (10.0)	8.9 (9.5)
17:00–20:00 h	-	28.8 (12.1)	30.5 (14.6)
20:00–24:00 h	-	7.1 (9.2)	8.2 (13.1)

Demographic data were missing in 32 participants for marital status and education, 35 participants for income, and 18 for race. ^1^ Participants were followed from pregnancy to postpartum. Data from 248 women were available both from pregnancy and postpartum, 117 only from pregnancy, and 18 only from postpartum. ^2^ Participants’ mean daily HEI scores (calculated by dietary recall day). ^3^ Including breakfast, lunch and dinner. ^4^ Including all eating occasions ≥ 50 kcal. ^5^ Participants skipping any main meal on any available recall.

**Table 2:** Associations of eating frequency with daily energy intake and HEI.

		Daily Energy Intake, kcal ^1^		Daily HEI, Total Score ^1^	
		Mean	95% CI	Mean	95% CI
Pregnancy	Main meals				
	Unadjusted model	210.9	132.6; 289.2 *	2.9	1.4; 4.4 *
	Adjusted model	300.7	199.3; 402.0 *	2.9	0.8; 5.0 *
	Eating occasions				
	Unadjusted model	138.2	111.2; 165.3 *	1.8	1.3; 2.4 *
	Adjusted model	161.6	129.6; 193.5 *	1.9	1.2; 2.5 *
Postpartum	Main meals				
	Unadjusted model	212.1	91.6; 332.6 *	3.9	1.7; 6.2 *
	Adjusted model	243.3	86.1; 400.6 *	3.1	−0.1; 6.2
	Eating occasions				
	Unadjusted model	169.6	125.5; 213.8 *	2.8	2.0; 3.6 *
	Adjusted model	146.4	91.4; 201.4 *	2.5	1.4; 3.6 *

HEI, Healthy Eating Index-2015. ^1^ Linear mixed regression was performed with REML, restricted maximum likelihood. *n* = 365 in pregnancy and *n* = 266 in postpartum. Adjusted model was adjusted for income, race, education, recall on weekday/weekend day (ref = weekday), weekly frequency of moderate/vigorous physical activity, eating less due to nausea (pregnancy models only), and breastfeeding duration (postpartum models only). * α < 0.05 indicates statistical significance.

**Table 3:** Associations of eating regularity with mean energy intake and overall HEI.

		**Mean Energy Intake, kcal/d** ^1^		**Overall HEI, Total Score** ^1^	
		**Mean**	**95% CI**	**Mean**	**95% CI**
Pregnancy	Meal skipping (ref = regular meal eating)				
	Unadjusted model	−190.0	−341.3; −38.6 *	−5.6	−8.3; −2.9 *
	Adjusted model	−243.4	−384.4; −102.3 *	−4.2	−6.7; −1.6 *
	Energy irregularity score (1 SD change)				
	Unadjusted model	24.8	−47.4; 97.1	−1.5	−2.9; −0.3 *
	Adjusted model	4.3	−64.1; 72.5	−0.9	−2.1; 0.3
Postpartum	Meal skipping (ref = regular meal eating)				
	Unadjusted model	−173.8	−358.1; 10.5	−7.6	−11.2; −4.0 *
	Adjusted model	−150.0	−327.3; 27.3	−6.2	−9.7; −2.7 *
	Energy irregularity score (1 SD change)				
	Unadjusted model	4.3	−79.1; 87.6	−2.4	−4.1; −0.7 *
	Adjusted model	1.3	−80.1; 82.7	−1.6	−3.2; 0.1

HEI, Healthy Eating Index-2015. ^1^ Linear regression was performed with FIML, full information likelihood ratio. *n* = 303 in pregnancy and *n* = 206 in postpartum. Adjusted model was adjusted for income, race, education, proportion of recalls on weekend, mean weekly frequency of moderate/vigorous physical activity, highest value reported for eating less due to nausea (pregnancy models only), and breastfeeding duration (postpartum models only). * α < 0.05 indicates statistical significance.

**Table 4:** Associations of intake timing patterns with mean energy intake and overall HEI.

		Mean Energy Intake, kcal/d ^1^		Overall HEI, Total Score ^1^	
		Mean	95% CI	Mean	95% CI
Pregnancy	Evening eating pattern				
	Unadjusted model	−0.6	−1.7 to 0.6	0.3	−0.9 to 1.4
	Adjusted model	−0.3	−1.4 to 0.8	0.3	−0.8 to 1.4
	Late morning/early afternoon eating pattern				
	Unadjusted model	−0.3	−1.4 to 0.9	−0.1	−1.3 to 1.0
	Adjusted model	−0.2	−1.3 to 1.0	−0.7	−1.8 to 0.5
	Early morning eating pattern				
	Unadjusted model	−1.5	−2.6 to −0.4 *	1.1	−0.0 to 2.2
	Adjusted model	−1.4	−2.5 to −0.3 *	1.0	−0.1 to 2.1
Postpartum	Evening eating pattern				
	Unadjusted model	−0.7	−2.4 to 1.0	2.4	0.7–4.0 *
	Adjusted model	0.2	−1.6 to 1.9	1.2	−0.5 to 2.9
	Late morning/early afternoon eating pattern				
	Unadjusted model	0.2	−1.2 to 1.6	0.4	−0.9 to 1.8
	Adjusted model	0.6	−0.8 to 1.9	−0.3	−1.6 to 1.0
	Early morning eating pattern				
	Unadjusted model	−0.8	−2.1 to 0.5	1.4	0.1–2.7 *
	Adjusted model	−0.7	−2.0 to 0.6	1.4	0.2–2.6 *

HEI, Healthy Eating Index-2015. ^1^ Intake timing patterns were derived by principal component analysis from percent of mean daily energy intake within time windows. ^1^ Linear regression was performed with FIML, full information likelihood ratio; *n* = 365 in pregnancy and *n* = 265 in postpartum. Adjusted model was adjusted for income, race, education, proportion of recalls on weekend, mean weekly frequency of moderate/vigorous physical activity, highest value reported for eating less due to nausea (pregnancy models only), and breastfeeding duration (postpartum models only). Results show a 1 SD change in pattern scores. * α < 0.05 indicates statistical significance.

## Data Availability

Data described in the manuscript, code book, and analytic code will be made available upon request pending application and approval.

## Abbreviations

HEI: Healthy Eating Index-2015; PCA, principal component analysis; PEAS, Pregnancy Eating Attributes Study; UNC, University of North Carolina.
